# Micro RNAs and the biological clock: a target for diseases associated with a loss of circadian regulation

**DOI:** 10.4314/ahs.v20i4.46

**Published:** 2020-12

**Authors:** Qianwen Ma, Genlin Mo, Yong Tan

**Affiliations:** 1 Gynecology department, Zhenjiang Hospital Affiliated to Nanjing University of Chinese Medicine (Zhenjiang Hospital of Traditional Chinese Medicine), Zhenjiang, China; 2 Reproductive medicine department, Affiliated Hospital of Nanjing University of Chinese Medicine, Nanjing, China; 3 Advanced manufacturing institution, Jiangsu University, Zhenjiang, China

**Keywords:** MicroRNAs, biological clock, circadian rhythm

## Abstract

**Background:**

Circadian clocks are self-sustaining oscillators that coordinate behavior and physiology over a 24 hour period, achieving time-dependent homeostasis with the external environment. The molecular clocks driving circadian rhythmic changes are based on intertwined transcriptional/translational feedback loops that combine with a range of environmental and metabolic stimuli to generate daily internal programing. Understanding how biological rhythms are generated throughout the body and the reasons for their dysregulation can provide avenues for temporally directed therapeutics.

**Summary:**

In recent years, microRNAs have been shown to play important roles in the regulation of the circadian clock, particularly in Drosophila, but also in some small animal and human studies. This review will summarize our current understanding of the role of miRNAs during clock regulation, with a particular focus on the control of clock regulated gene expression.

## Introduction

Circadian clock represents a ubiquitous internal mechanism that allows organisms to adapt to cyclic changes in temperature, light, and other environmental factors^1^. Circadian clock is a 24 hour cycle that is generated endogenously, and can be modulated by external cues including natural light and temperature^2^. Circadian clock determines the sleeping and feeding patterns of all mammals, including humans^3^. Accumulating evidence demonstrates that the disruption of normal circadian clock contributes to the progression of cardiovascular disease^4^, metabolic disorders^5^–^6^, chemoresistance^7^, and neurodegenerative diseases^8^. Several mechanisms of functional impairment due to an abnormal circadian clock have been proposed, including altered cell signaling, cellular metabolic changes, and inflammation6. Scientists has revealed mechanisms underlying circadian clock function through studying Drosophila. As a model organism, the study of circadian rhythm in drosophila was initiated in 1971 when scientists obtained the first mutant of the clock Per in Drosophila through the screening of chemical mutagens. This landmark study opened up a new era for the study of molecular mechanisms of circadian rhythm. Rhythmic behavior could be evaluated to the function of several clock genes that produce circadian oscillations in certain brain neurons, which finally adjust the behavior in a circadian manner^9^. On a molecular level, circadian rhythm (CR) is driven by a series of transcriptional and translational feedback loops that drive the cyclic expression of clock genes including clock and bmal1, per1-3 and cry1-2, as well as their translational products a BMAL1-CLOCK complex, PER1-3, and CRY1-2. The levels of these clock genes serve as markers of CR8,^10^,^11^. The suprachiasmatic nucleus (SCN), a region of the hypothalamus situated above the optic chiasm, is the core circadian clock^12^. The peripheral clocks are biological oscillators which can specifically regulate various physiological activities in different tissues by regulating the expression of downstream clock genes^13^. It is now recognized that many molecular mechanisms influence circadian gene expression including changes to the architecture of chromatin^14^, interactions with transcription factors^10^, post-transcriptional RNA modifications^3^, post-translational modifications^15^–^16^, and protein trafficking and degradation^7^,^17^–^18^. Circadian rhythm is a positive and negative feedback loop composed of major clock genes, such as Clock, Bmal1, Per and Cry. Clock and Bmal1 transcription factors belong to the positive feedback loop, while Per and Cry transcription factors belong to the negative feedback loop. The positive feedback loop promotes transcription of clock genes. The negative feedback loop inhibits transcription of clock genes^19^. Recently, alternative RNA splicing and microRNAs (miRNAs) have emerged as key players in clock regulation, raising the intriguing possibility that clock-controlled miRNAs contribute to disorders of the human circadian system. In this review, we provide an up-to-date overview of the role of miRNAs in circadian clock physiology, and discuss new methods to harness their ability to influence circadian physiology.

## MicroRNA discovery and synthesis

miRNAs are non-coding RNAs consisting of 21–25 nucleotides that act as regulators of gene expression in eukaryotic cells. In eukaryotes, miRNAs are synthesized from primary miRNAs in a 2-stage process by the action of two RNase III-type proteins termed Drosha, in the nucleus, and Dicer, in the cytoplasm. In recent years, significant advances have been made in our knowledge of miRNA processing in mammalian cells. The process is initiated when the genes for miRNAs are transcribed to a primary miRNA by RNA polymerase II. The primary miRNA is then processed in the nucleus to a precursor miRNA (pre-miRNA) of 70–100 nucleotides by Drosha. The pre-miRNA is exported to the cytoplasm via exportin-5 and further processed by Dicer into mature miRNAs. These are then loaded onto the Argonaute protein to produce the effector RNA-induced silencing complex (RISC).

miRNAs were first discovered in 1993 by Lee and co-workers in experiments utilizing the embryos of the nematode Caenorhabditis elegans (C. elegans)^20^. In these organisms, the downregulation of LIN-14 is essential to the progression of the first larval stage L1 to L2. LIN-14 downregulation was shown to depend on the transcription of a secondary gene termed lin-4. It was found that the transcribed lin-4 was not translated into a protein, but instead gave rise to two small RNAs (21 and 61 nucleotides long), the longer sequence forming a stem-loop and acting as a precursor for the shorter RNA. Later, the same group along with Wightman^21^ discovered that the smaller RNA had antisense complementarity to the 3′-UTR of lin-14 mRNA. This binding decreased LIN-14 expression at the protein level without influencing its mRNA abundance. These two studies together proposed the now accepted model wherein base pairing between multiple lin-4 miRNAs to complementary sites in the 3′-UTR of lin-14 mRNA leads to translational suppression, promoting the progression from L1 to L220-21. In 2000, it was discovered that a second miRNA, let-7, was required for the development of a later larval stage in C. elegans^22^. Importantly, homologues of this gene were then discovered in many organisms including human cells, revealing cross-species roles for miRNAs. In the period that followed, a large number of studies from multiple laboratories cloned miRNAs from humans, worms, and flies. The miRNAs share many features; they are 19 to 24 nucleotides in length, non-coding, and derive from a longer precursor with a stem-loop structure. Most of the identified miRNAs are evolutionarily conserved and display cell-type specificity^23^. To-date, multiple miRNAs have been discovered across numerous species of plants and animals. However, their biological significance often remains poorly defined and requires functional validation.

## miRNAs and their functional relevance

The importance of miRNAs is highlighted by the fact that animals which fail to express them do not survive^24^. Dicer silencing is the accepted method of miRNA inhibition, and leads to embryonic lethality in mice due to abnormal morphology of almost all organs^25^. The differentiation of embryonic stem cells (ESCs) is key to organ development and is modulated by miRNAs. In mice, Dicer silencing does not influence the formation of ESC colonies, but severely impairs their differentiation^25^–^26^. MiR-290 clusters that are expressed from a single transcript directly target the cell cycle suppressors p21 and LATS2, facilitating ESC G1-S phase progression^27^–^28^. The transcription factors Oct3/4, Nanog, and Sox^2^, are required for the maintenance of ESC pluripotency and bind to the promoter of miR-290 clusters in order to sustain their expression, thereby promoting self-renewal and maintaining pluripotency. In contrast, miRNA let-7 is a suppressor of pluripotency and inhibits miR-290^29^. In the differentiated state, let-7 is upregulated suggesting that its antagonistic effects promote ESC differentiation. miRNAs similarly regulate hematopoietic stem cells. MiR-125b is a miRNA that regulates inflammation and innate immunity by specifically promoting macrophage differentiation and activation through nuclear factor (NF)-κB pathway signaling^30^–^32^. Accordingly, the dysregulation of miR-125b has been reported in many cancers, including leukemia.

miRNAs have now been shown to play a major role in an array of cell types. Although the miRNA class of molecules is ubiquitously expressed, their specific spatial and temporal expression is regulated according to tissue type. Examples include the requirement for miR-273 during neuronal development^33^, the role of miR-1 in cardiogenesis by regulating Hand^2^ expression^34^, miR-133a and miR-27 that regulate myocyte proliferation^34^–^35^, miR-203 that is induced during stratification of mouse skin and controls basal to suprabasal transition^36^, miRNA-375 that regulates pancreatic development^37^, miRNA-430 that participates in neuronal development in zebrafish^38^, and miRNA-181b that inhibits IGF-1R expression and can repress glioma development^39^. These and emerging studies have unequivocally established the relevance of miRNAs to mammalian development and the regulation of important physiological processes.

Bahk YC40 showed a close correlation between biorhythm and suicidal ideation in patients with depression through investigation. The occurrence of suicidal thoughts in patients with morning depression was significantly less than that in patients with night depression. La Morgia^41^ also studied that the occurrence of biorhythm disorder is closely related to the occurrence and development of alzheimer's disease. To sum up, the disturbance of biological rhythm will cause the dysfunction of the body's nervous system, which can lead to the occurrence and development of a variety of neurological diseases.

## miRNAs and the control of circadian rhythms

### Drosophila studies

CR processes are driven by endogenous molecular clocks that regulate the expression of clock-controlled genes (CCGs). The transcription of CCGs is controlled by the rhythmic action of transcription factors and circadian alterations in chromatin^14^,^42^. In recent years, the importance of post-transcriptional regulation which alters the levels and phase regulation of CCG mRNA and protein expression in CR has emerged^3^,^33^,^42^–^45^.

Circadian clocks enable organisms to adapt to fluctuating environmental conditions. In this regard Garaulet and colleagues^46^ found that miR-124 could regulate the CR of Drosophila. During normal light/dark cycles, miR-124 loss-of-function mutations caused abnormal locomotor activity, leading to a loss of anticipatory capacity during morning/evening transitions. In addition, miR-124 mutants caused behavioral alterations in continual darkness. Anatomical and functional tests revealed a normal circadian pacemaker in miR-124 mutants, suggesting that miR-124 regulates clock output. After profiling the miR-124 interaction network, targets in the Bone Morphogenetic Protein (BMP) pathway were shown to correct the evening activity phase shift in continual darkness. It was therefore confirmed that BMP signaling drives specific circadian behaviors in miR-124 knock-out flies.

Chen^47^ also studied Drosophila of circadian rhythms. They found that mRNA-276a regulates molecular and behavioral rhythms by inhibiting expression of important CCGs. Dysregulation of miR-276a in clock neurons altered timeless expression and increased arrhythmicity in constant darkness. MiR-276a was shown to be light-regulated and its expression controlled by the transcriptional activator Chorion factor 2 (CF-2). Deletion of the miR-276a-binding sites in the timeless 3′-UTR led to elevated levels of timeless protein expression and enhanced arrhythmicity. Thus, this pathway contributes to more robust rhythms observd under light/dark conditions compared to constant darkness.

Sun^48^ continued to study Drosophila. They found that high levels of miRNA-279 and miRNA-996 in Drosophila regulated multiple biosynthetic processes. miRNA-279 deficiency could limit the synthesis of CO2 sensory neurons and regulate CR. miRNA-996 was found to localize adjacently to miR-279 and possess similar functionality.

Cusumano^49^ also found there was relationship between Drosophila and circadian rhythms. They revealed the role of miR-210 in regulating circadian rhythm outputs and guiding/remodeling PDF positive LNv branches in Drosophila, and suggested that miR-210 may have pleiotropic effects on the clock, light perception and neuron development.

Moreover, You^50^ studied Drosophila of biological rhythms. They founded miR-263b and miR-274 in detail and found that they both have the function of regulating behavior in adult astrocytes. Astrocyte-specific inhibition of miR-263b or miR-274 in adults significantly impaired circadian rhythm, but had no effect on the viability of glial or clock neuron cells. Knockdown of glial cells in two hypothetical miR-274 targets, CG4328 and MESK2, resulted in a significant decrease in rhythmicity. Homology between target genes of miR-274 and mammals suggests that its mechanism may be related to glial regulation of rhythms.

### Rat and mouse models

Although progress in the field had implicated miRNAs in development and disease, the expression and function of miRNAs in the nervous system was not as well characterized. In 2007, Chen and colleagues^51^ examined two brain-specific miRNAs located in the SCN, miR-219 and miR-132, for their ability to modulate CR. Their studies revealed that miR-219 was a target of the Clock/Bmal1 complex which exhibited CR dependent expression, and that in vivo knockdown of miR-219 lengthened the circadian period. They further showed that miR-132 was induced by photic cues that are dependent on the MAPK/CREB-signaling axis, which was found to modulate CCG expression, and attenuate the entraining effects of light. To address the mechanisms underlying these effects, they compiled a list of targets of miR-132 and miR-219 using prediction algorithms and speculated that miR-219 and/or miR-132 could influence the clock via alterations in cell excitability. When cultured cortical neurons were transfected with miR-219 or miR-132 and the effects on depolarization- and glutamate receptor-evoked Ca2+ responses assessed, miR-132 significantly potentiated K+, glutamate, and NMDA administration responsiveness, whilst miR-219 triggered a modest but significant attenuation of evoked responsiveness. Collectively, this study revealed that both light-responsive miR-132 and clock-regulated miR-219 influence membrane potential and cellular excitability.

Shende^52^ examined miR-142-3p for evidence of circadian expression in the SCN and regulation of its putative CCG target Bmal1 via specific binding sites in the 3’ UTR. They also assessed miR-142-3p overexpression-induced changes in the CR of Bmal1. Mutagenesis studies revealed that two independent miRNA recognition elements equally contributed to miR-142-3p-induced repression. Overexpression of miR-142-3p also abolished circadian variation and endogenous Bmal1 protein levels in vitro. This confirmed that miR-142-3p contributes to post-transcriptional modulation of Bmal1 and its contribution to CR.

Furthermore, Ding^53^ investigated the mechanisms of post-transcriptional regulation of CCGs through screening changes in miRNA levels in the pineal gland. miR-182 was found to target the 3′-UTR of clock and regulated Clock expression following hypoxic exposure of cultured pinealocytes. Zhang and colleagues47 found that miR-27b-3p exhibits rhythmic expression in the metabolic tissue of mice exposed to constant darkness. The expression of miR-27b-3p was induced in the livers of unfed and ob/ob mice and the oscillatory expression of miR-27b-3p could be reversed by restricted feeding, suggesting its contribution to regulation of the peripheral clock. The same study identifiedmal1 as a direct target of miR27b-3p explaining its role in CR and energy metabolism in the liver.

MiR-155 was also found to regulate biological rhythm via bmal1. In bone marrow, Bmal1 could inhibit the activation of NF-κB and inhibit the synthesis of miR-155, allowing rats to avoid septicopyemia in response to Lipopolysaccharide (LPS). Two miR-155 binding sites were identified in the 3′-UTR of Bmal1 and miR-155 binding inhibited Bmal1 expression. Cells lacking miR-155 had changes in CR and altered synthesis of an array of cellular factors that control the biological clock^54^.

Gao^55^ also studied circadian rhythms in the mouse models. They found that miR-17-5p, which had been previously implicated in tumor biology, also controlled CR. miR-17-5p was shown to be rhythmically expressed in synchronized fibroblasts and mouse SCN and inhibited the translation of clock by binding to its 3′-UTR. Clock also directly bound to the miR-17 promoter and enhanced its transcription and production suggesting a reciprocal relationship. Changes in Clock expression led to increased Cry1 expression and shortening of the free-running period in in vivo studies. Thus miR-17-5p was shown to play an important role in stabilization of the circadian-clock through indirect regulation of Cry1. In addition, Kiessling^56^ also revealed a strain and light-specific function of miR-132/212 in the circadian mechanism, suggesting that miR-132 and miR-212 are background-dependent circadian regulators.

Moreover, Yoo^57^ also found that Bmal1 mRNA and protein oscillation amplitude as well as Cry1 protein oscillation increased in Per2::LucSV mice, suggesting that rhythmic overexpression of Per2 could enhance the expression of core clock genes such as Per2.

### Human studies

Li^58^ identified that clock expression levels were elevated in high grade glioma tissue compared to low grade glioma and normal tissue. miR-124 was found to directly act upon clock and inhibit its expression. In glioma, the downregulation of miR-124 led to increased clock expression and reduced proliferation and migration of glioma cells. Clock was further found to regulate glioma cell proliferation and migration through NF-κB signaling^58^.

In addition, Han^59^ found that miR-34a reduced the likelihood of cholangiocarcinoma in human cells through its action on PER1. In cholangiocarcinoma, PER1 expression is decreased and periodical rhythm is lost. Cells that overexpress PER1 displayed reduced proliferation, decreased G2/M lag phase, and enhanced apoptotic induction. In vivo PER1 overexpression reduced tumor growth, proliferation, angiogenesis, and metastasis. Inhibiting miR-34a similarly promoted the metastasis of cholangiocarcinoma cells. This highlighted how manipulation of the molecular clockwork in humans may prove beneficial during the treatment of serious human diseases.

### Zebrafish studies

Using miRNA sequencing technologies, pineal-enhanced and light-induced miRNAs were identified by Ben-Moshe^60^. One such miRNA, miR-183, was shown to downregulate e4bp4-6 which regulated the rhythmic mRNA levels of aanat2, a key enzyme in melatonin synthesis. This genome-wide approach and functional characterization of light-induced factors indicated multi-level regulation of the circadian clock by light.

Cold shock can induce acute physiological stress responses and transcriptional changes in aquatic creatures. Hung^61^ revealed a role for miRNAs in acute cold responses through small RNA-seq analyses, identifying numerous differentially expressed miRNAs. Overexpression of Per2 resulted in partial recovery from cold shock. Subsequently the interaction of Per2 with miR-29b was identified, and this miRNA was also found to be cold-inducible. This study characterized the functional roles of CCGs during cold tolerance

## Other miRNAs related to CR

Other model systems of note that have revealed key circadian roles of miRNA genes including the identification of miR-206 as an important regulatory factor in mammalian skeletal muscle cells^62^. By controlling changes of amplitude and frequency, miR-206 influences gene expression and interrupts gene synthesis. Riley^63^ found that miR-125a-5p affected the stability of the Lfng transcripts in the anterior mesoderm of chickens, and its inhibition disrupted CR functionality. Chickens lacking miR-125a-5p expression also displayed abnormal embryonic cell division. Nocturnin, an adenase that is expressed downstream of the CR, acts to regulate metabolism in response to biological rhythm. Nocturnin was revealed as a target of miR-122, a liver specific miR-122 known to participate in lipid metabolism^64^.

## Summary and future prospects

Recently, miRNAs have emerged as significant players in CR timing, raising the possibility that clock-controlled miRNAs contribute to disorders of the human circadian timing system. Since the biochemical activity of an array of cell types and organs is shaped by our 24-hour CR, miRNAs impart circadian modulation over a wide range of behavioral and physiological processes. Indeed, rhythmic drive regulates up to 15% of the transcriptome. Dysregulation of miRNAs, and thus the clock, is implicated in the pathogenesis of many disorders ranging from hypertension to cancer. Therefore, new therapeutic approaches targeting miRNA expression and clock physiology are of major interest. A more comprehensive understanding of the underlying molecular mechanisms that modulate miRNAs involved in CR and give rise to organ-specific CR transcriptomes is now required. This information will allow us to unlock the utility of miRNAs as effectors of CR physiology and in the pathophysiology of clock related disease processes.

## Abbreviations

CR, circadian rhythm; SCN, suprachiasmatic nucleus; miRNAs, microRNAs; pre-miRNA, precursor miRNA; C. elegans, Caenorhabditis elegans; ESCs, embryonic stem cells; NF, nuclear factor; CCGs, clock-controlled genes; BMP, bone morphogenetic protein; CF-2, chorion factor 2; LPS, lipopolysaccharide
